# Continuity and discontinuity in Native American populations: Insights from ancient and modern mitochondrial DNA

**DOI:** 10.1590/1678-4685-GMB-2025-0248

**Published:** 2026-07-20

**Authors:** Gustavo Medina Tavares, Bastien Llamas, Rafael Bisso-Machado

**Affiliations:** 1 Universidade Federal do Rio Grande do Sul (UFRGS), Instituto de Biociências, Departamento de Genética, Programa de Pós-Graduação em Genética e Biologia Molecular, Laboratório de Evolução Humana e Molecular (LEHM), Porto Alegre, RS, Brazil.; 2 The University of Adelaide, School of Biological Sciences, Australian Centre for Ancient DNA, Adelaide, SA, Australia.; 3 Australian National University, John Curtin School of Medical Research, National Centre for Indigenous Genomics, Canberra, ACT, Australia.; 4 The Kids Research Institute Australia, Indigenous Genomics, Adelaide, SA, Australia.; 5 Universidade Federal de Ciências da Saúde de Porto Alegre (UFCSPA), Programa de Pós-Graduação Patologia, Porto Alegre, RS, Brazil.

**Keywords:** Ancient DNA, mtDNA, American continent, Native American, genetic continuity

## Abstract

Ancient DNA research has expanded dramatically in recent years, transforming reconstructions of how and when humans settled the American continent. In this review, we synthesize evidence for genetic temporal continuity and discontinuity in Native American population history using mitochondrial DNA (mtDNA) from ancient and contemporary individuals. We systematically surveyed studies indexed in PubMed, ResearchGate, and Google Scholar, extending earlier compilations with publications through October 2025. For each population, we compiled sample sizes, mtDNA haplogroup frequencies, geographic coordinates, and associated archaeological and chronological information. These data were used to map the spatial distribution of founding mtDNA lineages, examine regional trajectories of haplogroup frequencies, and characterize patterns of mitochondrial population structure. Geographic distance showed a statistically detectable but limited association with mtDNA differentiation. Time-ordered haplogroup frequency series revealed region-specific dynamics, including long-term persistence of maternal lineages as well as marked episodes of turnover, some coincident with major cultural transitions. Genetic data were obtained from 315 studies. The dataset for contemporary populations included 23,315 individuals from 322 populations, while ancient DNA data included 4,192 individuals associated with 211 archaeological populations. Together, these data provide a comprehensive synthesis of mtDNA-based temporal and spatial patterns relevant to the peopling of the American continent.

## Introduction

### Peopling of the American continent

The peopling of the American continent is a complex and fascinating chapter in human evolutionary history. Genetic evidence indicates that Native Americans originated from a Beringian ancestral population that expanded into the continent from Eastern Eurasia at least 16,000 years before present (yBP), with subsequent episodes of contact and admixture among different ancient groups (reviewed in *e*.*g*., Mulligan and Szathmáry, 2017; Bisso-Machado and Fagundes, 2019; Hoffecker et al., 2019; Pinotti et al., 2019; Hünemeier et al., 2025). From the early 1990s, uniparental genetic markers became the primary genetic resource to investigate evolutionary relationships among Native American populations. Early studies revealed a restricted set of “founding” lineages, distinguishing Native Americans from their Asian relatives while confirming their close affinity (*e*.*g*., Schurr et al., 1990; Torroni et al., 1993; Bailliet et al., 1994; Pena et al., 1995; Santos et al., 1996). They also ignited debates around the timing of the initial settlement of the American continent based on molecular clock estimates (*e*.*g*., Forster et al., 1996; Underhill et al., 1996; Bonatto and Salzano, 1997 a , b; Bianchi et al., 1998).

The arrival of Europeans and the forced displacement of Africans from the late fifteenth and sixteenth centuries onward had a profound impact on Native populations, introducing new pathogens and triggering complex immune adaptations shaped by multifactorial causes (Collen et al., 2022). Contact with Europeans and Africans resulted in patterns of genetic and cultural continuities and discontinuities in Native American populations across the continent (O’Fallon and Fehren-Schmitz, 2011; Lindo et al., 2018 a ; Hofman and Keehnen, 2019; Nieves-Colón, 2022) themes that are central to this review.

### Mitochondrial DNA (mtDNA)

The first complete human mitochondrial genome was published in 1981 (Anderson et al., 1981). Early population surveys nonetheless relied on restriction fragment length polymorphisms (RFLPs) (Denaro et al., 1981; Merriwether et al., 1991). By the late 1980s and early 1990s, targeted sequencing of the control region (D-loop) became routine (Vigilant et al., 1989, 1991; Horai and Hayasaka, 1990). In 1999, the original Cambridge Reference Sequence was re-examined, corrected, and released as the revised CRS (rCRS), providing a stable benchmark for reporting mitochondrial DNA (mtDNA) variation (Andrews et al., 1999). Subsequent advances in genotyping and high-throughput sequencing enabled whole mitochondrial genome datasets (*e*.*g*., Tamm et al., 2007; Achilli et al., 2008; Fagundes et al., 2008), which refined the classic set of five founder haplogroups (A, B, C, D, X) into more than 15 founder sub-haplogroups (Achilli *et al*., 2013).

### Ancient DNA

Building on Pääbo’s pioneering recovery of DNA from ancient human remains (Pääbo, 1985) and the first PCR-based mtDNA sequences from a ≈7,000-year-old brain (Pääbo *et al*., 1988), the subsequent systematic recovery of molecular data from ancient humans initially leveraged uniparental markers-particularly mtDNA-because its short length and high copy number made it well suited to aDNA studies *(e*.*g*., Stone and Stoneking, 1993; Monsalve et al., 1996). These early studies demonstrated that mitochondrial genetic information could be retrieved from archaeological specimens, laying the foundation for integrating ancient DNA (aDNA) into reconstructions of Native American genetic and demographic history.

A defining challenge in aDNA research is the chemical instability of DNA molecules after death. Unlike metabolically active tissues, which repair molecular damage, ancient remains accumulate lesions over time (Lindahl, 1993; Willerslev and Cooper, 2005). Environmental factors such as temperature fluctuations, pH, oxygen exposure, and water infiltration accelerate degradation through hydrolysis, oxidation, and alkylation reactions (Austin et al., 1997; Telles and Diniz Filho, 2004; Hansen et al., 2017). Thermal history is particularly critical: permanently frozen environments are conducive to long-term DNA survival, whereas tropical and even temperate climates often yield highly fragmented DNA. Additionally, the amount of endogenous DNA in archaeological remains is often extremely low (frequently less than 1%) (Hansen *et al*., 2017), making stringent laboratory protocols and selective sampling from dense skeletal elements (such as petrous bones and teeth) preferable for success (Pinhasi et al., 2015; Harney et al., 2021). Advances in dedicated clean-room facilities, improved DNA extraction techniques, and library preparation protocols have progressively expanded the range of viable samples and geographic regions accessible to aDNA research throughout continental America (Llamas et al., 2016; Posth et al., 2018; Nägele et al., 2020; Fernandes et al., 2021; Ferraz et al., 2023; Nakatsuka et al., 2023).

### Continuity and discontinuity

Mitochondrial DNA (mtDNA) is a maternally inherited, largely non-recombining genome that accumulates mutations along maternal lineages, giving rise to well-defined haplogroups that are widely used to reconstruct population history and evaluate genetic continuity across time (Pakendorf and Stoneking, 2005; Hernández, 2023). Because these haplogroups represent stable maternal lineages that can be tracked across generations and compared among populations from different temporal contexts, mtDNA has become a particularly useful marker for investigating patterns of population continuity and change through time. Population continuity broadly refers to situations in which a population preserves elements of its identity across time, a pattern often inferred from genetic similarity between samples collected at different periods and from evidence of limited external gene flow (McKenna et al., 2024). In genetic studies, this concept is commonly expressed as genetic continuity, referring more specifically to the persistence of genetic lineages or allele frequencies through time. In mitochondrial DNA studies, continuity is typically inferred from the persistence of maternal lineages within a given region across time. Conversely, we use the term genetic discontinuity in a strictly operational sense, referring to meaningful changes in the relative frequencies or presence/absence of mtDNA haplogroups through time within a given region, rather than to genome-wide discontinuity or complete population replacement.

Illustrative examples from the literature include Wari-post-Wari continuity in the Ancient Andes (Kemp et al., 2009); the persistence of the rare subclades A2ag and A2ah from individuals dated to ~5,000 BP to present-day Indigenous populations from the north coast of British Columbia (Cui et al., 2013); approximately 1,300 years of continuity of multiple lineages (including A2w1, A2ad, A2ap, A2u, A2r, A2m, A2g, and B2l) between ancient Maya individuals and present-day Maya from Tixcacaltuyub (Barquera et al., 2024); and ~1,000 years of continuity of the rare lineage B2y1 between ancient and present-day Picuris (Pueblo) populations in the southwestern United States (Pinotti et al., 2025).

In turn, examples of mitochondrial discontinuities are relatively rare in the literature, although they can be inferred from temporal changes in the composition and diversity of maternal lineages. For example, rapid changes in maternal lineage composition have been associated with cultural turnovers in the Central Andes during the Middle Horizon period (Fehren-Schmitz et al., 2014). Additional evidence of disruption comes from southern Brazil and Uruguay, where contemporary Native American groups retain less than 5% of the pre-contact mitochondrial variation, while surrounding admixed populations appear to act as genetic reservoirs of lineages that may have disappeared locally among Indigenous groups (Tavares et al., 2019). At a broader continental scale, surveys of ancient mitochondrial genomes also suggest a substantial loss of maternal lineages following European colonization across the Americas (Llamas et al., 2016).

Although mtDNA captures only the matrilineal record and is sensitive to drift and post-contact bottlenecks, the merging of contemporary and ancient mtDNA has also been instrumental in reconstructing continent-wide demographic history. Coalescent-based approaches applied to control-region and mitogenomic data have been used to identify historical expansions and contractions (*e*.*g*., Tamm et al., 2007; Achilli et al., 2008; Fagundes et al., 2008; Kitchen et al., 2008; Soares et al., 2009; O’Fallon and Fehren-Schmitz, 2011; Llamas et al., 2016). Phylogeographic analyses that map the spatial distribution of founding sub-haplogroups further resolve dispersal corridors (coastal versus interior), latitudinal diversity clines, and regional pulses of movement linked to cultural transitions (*e*.*g*., Tamm *et al*., 2007; Perego et al., 2009).

### Database

Bisso-Machado and Fagundes (2021) assembled the most comprehensive collection of uniparental markers for Native peoples of the American continent so far, integrating mtDNA and Y-chromosome data from both modern and ancient populations. Their analyses revealed that most genetic variation is found within rather than between populations, with weak evidence of isolation by distance at the continental scale. Clear asymmetries between maternal and paternal markers highlight sex-biased admixture after European contact, while aDNA data document cases of long-term continuity as well as striking discontinuities, such as haplogroup turnover in the Arctic and Central America. Together, these findings illustrate how uniparental markers capture both the resilience and the transformation of Native American lineages through time.

Building on the comprehensive compilation of mitochondrial data by Bisso-Machado and Fagundes (2021), this review extends the scope by incorporating studies published since 2021 and recovering pre-2021 works overlooked in the earlier study, integrating ancient and contemporary mtDNA to examine patterns of genetic continuity and discontinuity across the American continent. Beyond updating the empirical record, we combine continent-wide synthesis with formal analyses of population structure and multiple explanatory frameworks, allowing us to quantify the limited contribution of geographic distance, linguistic affiliation, and ecoregional context, and to identify region-specific temporal dynamics. Because our analyses are based on haplogroup frequencies rather than full mitochondrial sequences, they capture broad lineage-level patterns rather than finer sub-haplogroup resolution and thus emphasize population-scale trends over lineage-specific microdynamics that can guide future studies targeting sub-haplogroup resolution, full mitochondrial genomes, and formal demographic modeling in regions where continuity or turnover is most strongly suggested. This framework highlights not only the persistence of maternal lineages across time and space but also recurrent episodes of lineage loss and reorganization, offering new insights into Native American population history before and after European colonization.

## Material and Methods

### Data collection

We extended the dataset from Bisso-Machado and Fagundes (2021) with publications dated December 31, 2020, to October 5, 2025, identified via PubMed, ResearchGate, and Google Scholar. We screened titles/abstracts using combinations of the terms [mitochondrial DNA, mtDNA, haplogroup, Native American, Amerindian, Indigenous, ancient DNA, archaeological, and Americas] and we performed backward citation searches from eligible studies. We also identified pre-2021 studies that were not included in the previous review and have now been incorporated into the present work. During data curation, we detected duplicate entries in the previously published dataset (Bisso-Machado and Fagundes, 2021). The database compiled here incorporates this quality-control step, ensuring that duplicate records were removed. In a few cases, we provisionally retained entries because the original reports did not clearly indicate prior analysis of the same samples or because delineating populations is inherently problematic in this context (see Van Arsdale and Nelson, 2025). 

### Data treatment

For all populations, we recorded sample size, mtDNA haplogroup frequencies, geographic coordinates, linguistic classification, and ecoregional context (Table S1-S2). Archaeological and chronological information was included exclusively for ancient DNA samples (Table S2). Ancient samples were assigned to archaeological populations as in the original publications, which generally considered if the samples came from the same site and if they can be identified as belonging to the same material cultural tradition. Linguistic classification followed Greenberg and Ruhlen (2007), Eberhard *et al*. (2025), and Hammarström et al. (2025). Ecoregions were classified according to Bailey’s Ecoregions of the World (Bailey, 1989).

Native American mtDNA lineages were mapped to the founder set of haplogroups A2, B2, C1b, C1c, C1d, D1, and the less frequent C4c, D4h3a, and X2a under current nomenclature. To ensure comparability with prior literature, we analyzed frequencies using the collapsed set A, B, C, D, and X. All analyses were performed based on haplogroup frequencies to include as many populations as possible. Because haplogroup X was incompletely surveyed in older datasets, we merged X into the “Other” category in the primary analyses. In addition, all mtDNA haplogroups reported for these populations that are not of Native American origin (e.g., African or West Eurasian lineages introduced after European contact) were grouped under the category “Other” (Table S1-S2).

Contemporary populations were defined as in source publications. Archaeological populations were aggregated at the level of site and period (or clearly defined cultural phase) to reduce temporal averaging. Chronological information was taken as reported (calibrated or uncalibrated); where both were available, calibrated ages were used. Individuals with uncertain cultural or chronological assignments were excluded from population-level summaries unless the original authors provided a justified grouping.

### Data analysis

Two sets of continent-wide maps were generated: (i) sampling locations overlaid on Bailey’s ecoregional domains for the American continent (including Greenland and the Bering region), and (ii) interpolated surfaces of mtDNA haplogroup frequencies (A, B, C, D, X, and Other) to visualize broad-scale spatial structure. All geoprocessing and map generation were performed in R v.4.4.2 (R Core Team, 2024) within RStudio v.2024.12.1.563 (Posit Team, 2025). The packages *readr*, *dplyr*, and *tidyr* (Wickham *et al*., 2023, 2024a, b) were used for data import and manipulation; *sf* (Pebesma, 2018) to handle spatial objects; and *rnaturalearth/rnaturalearthdata* (Massicotte and South, 2023; South *et al*., 2024) to retrieve basemap layers. For the sampling map, we imported Bailey’s domains shapefile (Bailey, 1989; UNEP-WCMC, 2020) and classified polygons into his four domain levels (Dry, Humid Temperate, Humid Tropical, and Polar). For the frequency maps, we applied inverse distance weighting (IDW) interpolation using *gstat* (Gräler et al., 2016). All layers were projected in geographic coordinates (EPSG:4326) to ensure compatibility with reported sampling coordinates (latitude/longitude). Maps were drawn with *ggplot2* (Wickham, 2016), and cartographic elements (scale bars and north arrows) added using *ggspatial* (Dunnington, 2023).

To examine temporal changes in haplogroup composition, both ancient and present-day mtDNA data were compiled into regional time-series using 100-year temporal bins, and haplogroup frequencies were estimated for each time interval. Contemporary populations were included as the final time bin (time 0), representing aggregated present-day datasets for each region. All haplogroup frequencies and percentages reported in the text were derived from temporally aggregated summaries of the mtDNA datasets provided in Tables S1 (present-day) and S2 (ancient). The packages *readr*, *tidyr*, and *dplyr* were again used for data import, manipulation, and calculation (Wickham *et al*., 2023, 2024a, b), and all plots were generated with *ggplot2* (Wickham, 2016), with panels faceted by subcontinental-scale groupings (North America, Central America, the Caribbean, and South America) and sample size represented as a grey shaded area in the background. 

Because modern sample sizes greatly exceed those of aDNA and would otherwise dominate the visualization, the sample-size track was displayed using a region-normalized, power-compressed scaling (with raw N values retained as right-side labels) to improve the visibility of temporal variation in aDNA sample sizes. Additionally, beyond the four subcontinental-scale units, we highlight and discuss a selected set of geographic regions (the Caribbean, the Arctic, the ancient Andes, and Brazil) to avoid exhaustive coverage of all American areas. Throughout the manuscript, the definition of “region” is intentionally scale-dependent, reflecting the different spatial and historical dimensions addressed in each analysis, rather than a single fixed regionalization scheme. Within this framework, Brazil is treated as an aggregated macro-demographic unit rather than as a homogeneous biogeographic region, allowing broad temporal trends in mtDNA lineage representation to be contrasted with those observed in other continental-scale units. Accordingly, point estimates shown for Brazil are intended to summarize large-scale patterns and do not imply internal genetic or regional homogeneity.

We also computed AMOVA and pairwise FST in Arlequin 3.5 (Excoffier and Lischer, 2010) using haplogroup frequency data from present-day populations, applying the Weir-Cockerham estimator (Weir and Cockerham, 1984) rather than Φ-statistics based on molecular distances. To evaluate regional patterns of mitochondrial genetic structure, we first performed hierarchical AMOVA with populations nested within four broad American subcontinents (North America, Central America, the Caribbean, and South America). In this framework, differentiation was partitioned among subcontinents, among populations within subcontinents, and within populations.

To further assess regional heterogeneity, we also conducted subcontinent-specific AMOVAs, estimating genetic differentiation among populations separately within each subcontinent. We restricted the AMOVA to these four subcontinental groups, as extensive analyses employing alternative linguistic and ecoregional partitions were already presented by Bisso-Machado and Fagundes (2021). In addition, only populations with sample sizes n ≥ 10 were included in the interpolated maps, population structure analyses (AMOVA, pairwise F_ST_), Mantel tests, and MRM analyses, thereby reducing stochastic effects associated with small sample sizes.

Geographic coordinates (longitude-latitude, decimal degrees, WGS84) were used to compute great-circle geographic distances (km) under the Haversine formula, implemented in the *geosphere* package (Hijmans 2024). The correlation between genetic and geographic distances was first assessed through Mantel tests (Mantel, 1967; 10,000 permutations) using the *vegan* package (Oksanen *et al*., 2025). Mantel tests were performed both at the continental scale and separately within each subcontinent.

To quantify the independent contribution of geography, linguistic, and ecoregional context, we applied the multiple regression on distance matrices (MRM) with 10,000 permutations using the *ecodist* package (Goslee and Urban, 2007). MRM analyses were conducted at the continental scale and, where sample size permitted, at the subcontinental level. Predictor matrices included geographic distance, linguistic family dissimilarity based on the classification of Eberhard *et al*. (2025), and ecoregional dissimilarity. Linguistic and ecoregional variables were implemented as binary dissimilarity matrices (0 = same category; 1 = different category). Thus, pairs of populations belonging to the same linguistic family or occupying the same ecoregion were coded as 0, whereas populations belonging to different linguistic families or ecoregions were coded as 1. This categorical approach was adopted because no broadly accepted framework currently allows linguistic or ecoregional differences to be expressed as continuous quantitative distances at the continental scale considered here. Model performance was assessed using the coefficient of determination (R²). Both log-transformed and untransformed geographic distances were evaluated, as the logarithmic transformation stabilizes variance and reduces the disproportionate influence of extreme long-range comparisons. Mantel and MRM analyses were performed in R 4.4.2 (R Core Team, 2024).

## Results

### General data overview

The complete dataset comprised 27,507 individuals (Table S1, Table S2), of whom 84.8% were modern samples and 15.2% were ancient. This pattern contrasts with the distribution of study types: of the 315 studies included here, 56.4% analyzed ancient DNA, 42% modern DNA, and 1.6% both. Interestingly, for the first time across our group reviews (Bisso-Machado et al., 2012; Bisso-Machado and Fagundes, 2021), ancient Native American samples outnumbered modern Native American mtDNA samples among newly compiled data (1,084 vs. 746), accounting for 59.2% of new additions. The new data came from 42 studies; of these, four contributed only modern mtDNA and 39 contributed ancient mtDNA (see Tables S1-S2). Thirty-one of these studies were published from 2021 onward. Barquera et al. (2024) and Pinotti et al. (2025) contributed to both types. Two studies had already been cited in Bisso-Machado and Fagundes (2021), but not all of their data had been included (Shimada et al., 2005; Posth et al., 2018). No new modern mtDNA datasets were generated for the Caribbean; by contrast, ancient data increased substantially in the Caribbean (≈270%) and Central America (77.8%), and more modestly in South America (28.7%) and North America (25%).

North America accounts for the largest volume of newly generated ancient mtDNA datasets (37.2%), followed closely by South America (36.3%), with the Caribbean contributing 23.9%, and Central America lagging far behind (2.6%). Mexico (21.2%), the United States (15.1%), Peru (12.5%), and Dominican Republic (11.7%) are the countries with the most new ancient data. In contrast, South America continues to contribute the vast majority of newly generated modern mtDNA datasets (89.1%), with major contributions from Bolivia (63.9%), Peru (13.3%), and Ecuador (9.4%), followed by a smaller proportion from Brazil (2.5%). Interestingly, despite the well-documented ethical challenges and trust issues between U.S. genetic researchers and Native American communities (Garrison, 2013), new contemporary Indigenous samples from the United States have recently become available (Pinotti et al., 2025), representing 1.7% of the new data, with the remainder coming from Mexico (9.1%). However, modern mtDNA data severely declined across all regions compared to other decades, which may reflect increasing challenges in establishing collaborations with Native American communities and a shift in emphasis toward whole-genome research.

### Sample distribution

**Figure 1 -:**
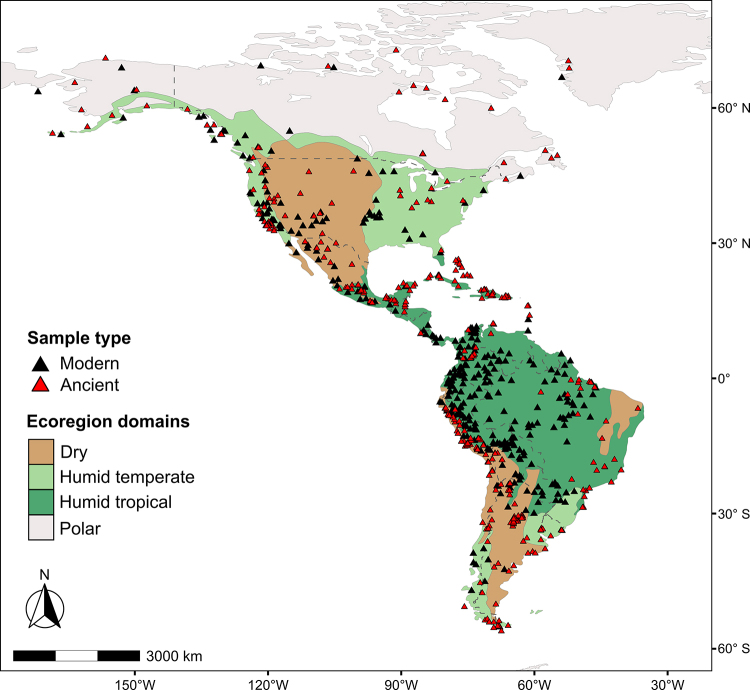
Geographic distribution of modern and ancient samples. Ecoregional domains are shown following Bailey (1989) to provide environmental context on the climatic and ecological settings in which Native American populations live or where archaeological sites are located. Dashed lines indicate modern political boundaries for geographic reference. Black triangles denote modern mitochondrial DNA samples; red triangles denote ancient DNA samples. All information about modern and ancient samples is provided in Tables S1-S2.

The sample distribution map (Figure 1) clearly illustrates the substantial representation of contemporary mtDNA samples from South America (56.2%), predominantly concentrated in Pacific-Andean and Amazonian regions, with notable contributions from Colombia, Peru, and Brazil. North America follows (39.5%), largely represented by the United States. Central America is third (4%) and the Caribbean contributes minimally (0.2%).

Ancient DNA samples appear to cover several American subcontinents but are in fact dominated by three countries-the United States, Peru, and Mexico, with important presence of Argentina and Chile. North America presents the largest proportion of ancient samples (48.2%), with the majority from the United States (27.4%, primarily in the western half) and Mexico (15.1%). The Arctic remains sparsely sampled overall but nonetheless exhibits broader coverage than several countries. 

South America also accounts for a large fraction of the total (41.8%), though heavily concentrated in Peru (17.8%), followed by Argentina (8.2%) and Chile (7.2%). Brazil (≈3%) has substantially increased its number of sampled sites, notably through Sambaqui (shell-mound) societies, yet it still lags behind several smaller countries. Central America continues to be the least sampled subregion (1.5%). The Caribbean has recently entered the aDNA map (8.5%), with major representation from the Dominican Republic (3.6%), Cuba (1.7%), and Puerto Rico (1.6%). In total, modern samples originate from 22 countries, whereas ancient DNA samples derive from 24 (Table S1, Table S2). Additionally, modern Native American samples originate from 437 distinct locations (Figure 1; Table S1), of which 63.3% lie in humid tropical ecoregions, 18.8% in humid temperate ecoregions, 14.9% in dry ecoregions, and only 3.0% in polar environments. Finally, aDNA derives primarily from archaeological sites in humid tropical (≈39.3%) and dry (≈34.8%) ecoregions (Figure 1; Table S2).

### Current haplogroup distribution

**Figure 2 -:**
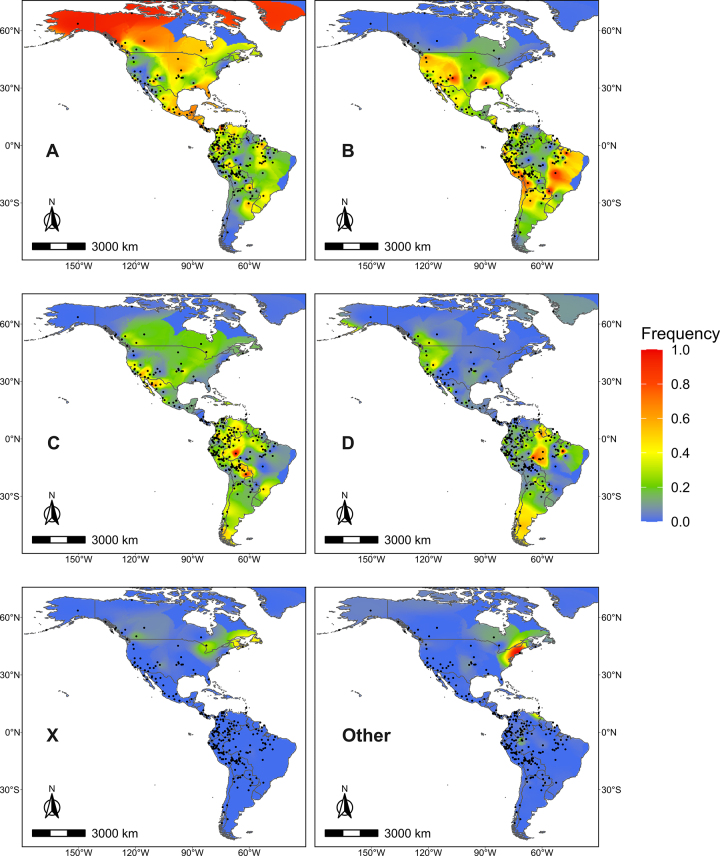
Spatial interpolation of mitochondrial haplogroup frequencies among Native American populations. The map shows the interpolated spatial distribution (Inverse Distance Weighting, IDW) of mitochondrial haplogroup frequencies (A, B, C, D, X, and Other) across the American continent. The color gradient represents the proportional frequency of each haplogroup in modern Native American groups, interpolated according to their geographic coordinates. Black circles indicate the locations of the sampled populations.

Haplogroup frequency distributions differ markedly across the four American subcontinents (Figure 2). Haplogroup A is extremely frequent in Arctic populations and in specific areas along the Ecuador-Peru border and northern Colombia, and it is generally common across the northern half of North America. Haplogroup B is very frequent among Native American groups in several regions of Brazil (*e*.*g*., the Central Plateau, the Paraguay border, and the Pará-Maranhão frontier) and is also prominent in the Andean corridor between Peru and Bolivia, as well as across the western half of the United States and portions of the southern and Midwestern regions. Haplogroup C is concentrated in western Amazonia (Brazil), in Bolivia (near the Brazil-Paraguay border), and along the western Mexico-United States frontier. Haplogroup D is notably more frequent in both the lower and upper stretches of the Brazilian Amazon, including the eastern margin, and across the southern cone (Argentina and Chile). Haplogroup X is most prominent in the northeastern United States and adjacent Atlantic Canada. The northeastern United States also shows the highest number of Native American groups reported to carry lineages outside the four major founding haplogroups (A-D). Importantly, in older datasets with limited resolution, such “non-ABCD” calls may reflect either bona fide Native American X lineages (when X-defining markers were not typed or were not reported) or post-contact admixture and, therefore, cannot be interpreted unambiguously. By contrast, the Garifuna of Saint Vincent and the Grenadines and the Santa Rosa First Peoples Community (FPC) of Trinidad and Tobago display numerous clearly non-Native haplogroups (Table S1), reflecting substantial admixture in the region with a strong African component.

### Continuities and discontinuities

*American subcontinents*

**Figure 3 -:**
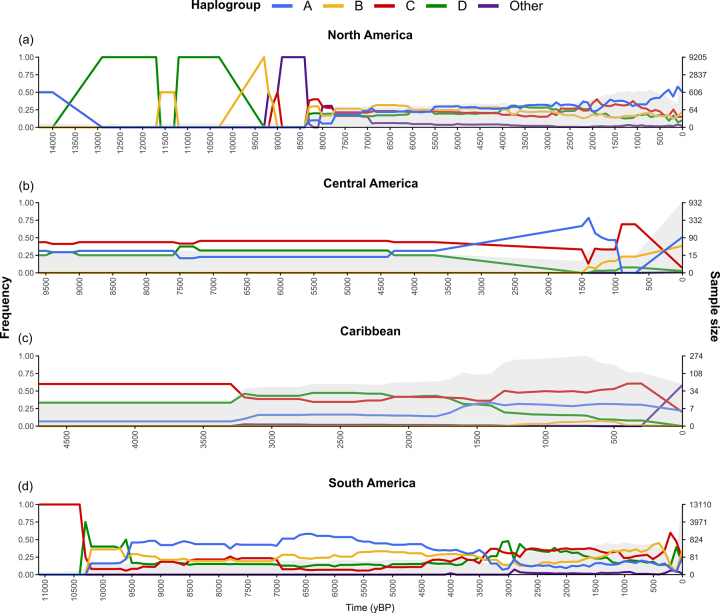
Temporal changes in mtDNA haplogroup frequencies across the American continent. Panels: (a) North America; (b) Central America; (c) Caribbean; (d) South America. Lines show the relative frequencies of haplogroups A, B, C, D, and Other (including X when available). The final interval (0 yBP) represents aggregated contemporary populations. Grey shading shows sample size through temporal bins on a compressed power-scaled axis (λ = 0.2), normalized within each subcontinent; right-side labels report raw sample sizes (N). Time is in years before present (yBP). Note that the time and sample-size axes are scaled independently in each panel.

The temporal variation in mtDNA haplogroup frequencies (Figure 3) reveals substantial shifts in lineage composition across the subcontinents of the American continent, although it does not provide a formal test of population replacement. These patterns are shown as point estimates and are intended as descriptive summaries of heterogeneously published data, rather than inferential tests of population continuity or stability. The timeframe spans from 14,300 years ago to the present. In this context, and given the exclusive use of mtDNA data, we use the term genetic discontinuity in a strictly operational sense, referring to meaningful changes in the relative frequencies or presence/absence of mtDNA haplogroups through time within a given region, rather than to genome-wide discontinuity or complete population replacement. While sample size appears to be a significant factor influencing mtDNA haplogroup composition, additional variables such as regional trends in archaeological research and potential local population replacements may also contribute to the observed patterns. These temporal trajectories may be characterized by alternating phases of apparent continuity, during which haplogroups persist with relatively stable frequencies, and phases of apparent discontinuity, when abrupt frequency shifts suggest historical or demographic inflection points in maternal lineage representation.

In the North American subcontinent (Figure 3, panel a), the class encompassing haplogroup X (“Other”) declines sharply between ≈7,800 and 6,900 yBP and remained low thereafter (6.1-0.5%) until its modern frequency (2.2%). Around the same time, haplogroup A begins a sustained rise, becoming the dominant lineage since 1,000 yBP until today (50.6%). Haplogroup D was prevalent in the oldest samples and remained stable for a long period between 8,300 and 400 yBP, with increasing importance between 4,000 to 3,000 (21.9-30.1%), then gradually decreasing to the lowest contemporary value among the major lineages (10.0%). Haplogroup B peaks in deeper time (≈11,600-11,300 yBP and 9,300 yBP) and then stabilizes near its modern level (22%), while haplogroup C follows a similar trajectory but importantly peaking between 1,900 and 1,100 yBP. After European arrival (≈500 yBP - present), B, C, and Other remain relatively unchanged, whereas A increases further and D continues to decline. 

Moving southward, the Central American subcontinent shows a different temporal pattern (Figure 3, panel b). Haplogroup C maintains the highest frequencies until ≈3200 yBP, then declines, reaching a historical maximum between ≈900 and ≈700 yBP (69.2%) before a steep drop near the onset of the colonial era, culminating in a low modern frequency (7.2%). Haplogroup A remains relatively stable until ≈3,300 yBP, then expands sharply to dominate by ≈1,400 yBP (78.3%), followed by a pronounced decrease and a later resurgence during the colonial period; it is currently the most frequent haplogroup (51.0%). Haplogroup B is rare prior to ≈1,500 yBP but rises afterward, particularly after ≈1,300 yBP, becoming the second most common lineage today (38.4%). Haplogroup D, by contrast, is more abundant in deeper periods and declines steadily from ≈3,700 yBP to near absence in recent times (2.6%).

In the Caribbean (Figure 3, panel c), where the genetic record becomes more robust after ≈3,300 yBP, haplogroup D predominates initially (3,200 - 1,800 yBP) but declines abruptly from ≈1,400 yBP onward and is absent from the modern dataset. Haplogroup B remains scarce throughout and is likewise absent today. Haplogroup A begins increasing around ≈3,300 yBP, reaching substantial frequencies between ≈1,600 and ≈300 yBP, remaining high during the colonial period and being the most frequent native lineage at present (21.8%). Haplogroup C remained substantially present across much of the temporal sequence, from early settlement to late Holocene, now matching A in frequency (20.0%). Non-native haplogroups constitute the majority in modern samples (58.2%), consistent with extensive admixture and the demographic collapse of Native American populations.

Finally, in South America, continuity and turnover also seem to alternate through time (Figure 3, panel d). Haplogroup A rises sharply after ≈9,600 yBP and dominates until ≈3,400 yBP. Haplogroup D contributes substantially during the early Holocene (≈10,300-9,600 yBP) and again between ≈3,100 and ≈1,500 yBP. Haplogroup B is well represented in the oldest samples and maintains considerable frequencies through the middle Holocene, becoming the leading lineage from ≈1200 to ≈300 yBP. Haplogroup C likewise is present in the oldest samples and shows enduring presence from ≈3,400 to ≈100 yBP, including prominent occurrences in *Sambaqui* from Jabuticabeira II and in *Botocudo* remains from southern and eastern Brazil, respectively. In present-day South America, all four native haplogroups remain well represented (A = 23.6%, B = 32.9%, C = 24.2%, D = 17.4%). These trends should be interpreted cautiously, as they may reflect a combination of temporal change, uneven sampling, and spatial aggregation of distinct populations within each time interval.

*Arctic*

**Figure 4 -:**
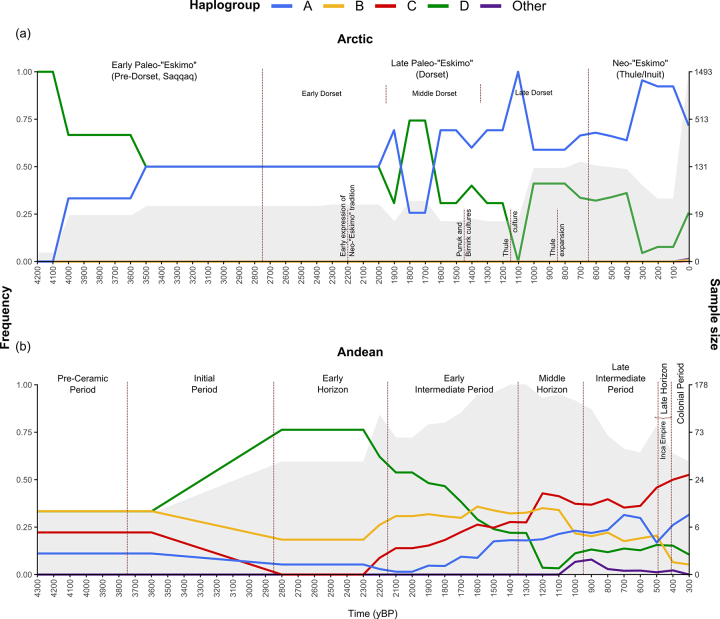
Temporal changes in mtDNA haplogroup frequencies across Arctic and Andean cultural sequences. Panel (a) shows haplogroup distributions through successive cultures in the Circumpolar region. Time intervals are based on Raghavan et al. (2014). Panel (b) displays the corresponding distributions across Andean archaeological periods. Lines represent the relative frequencies of haplogroups A, B, C, D, and Other (including X when available). The final interval (0 yBP) represents aggregated contemporary populations. Grey shading shows sample size through temporal bins on a compressed power-scaled axis (λ = 0.2), normalized within each region; right-side labels report raw sample sizes (N). Time is in years before present (yBP). Note that the time and sample-size axes are scaled independently in each panel.

Our analysis using only ancient and present-day Arctic populations with identified cultures (*Saqqaq, Aleut, Dorset, Thule,* and *Sadlermiut*) or population labels (*Aleut, Inuit*/*“Eskimo”*) shows that haplogroups A and D alternated as the most frequent from the earliest samples through the present (Figure 4, panel a), reaching parity between ≈3,500 and 2,000 yBP. From 1,600 to 100 yBP, haplogroup A remained highly predominant (58.9-95.0%) and still dominates present-day Arctic populations (71.6%), followed by D (25.8%), with residual frequencies of B (0.8%), C (0.3%), and Others (1.5%).

*Haplogroup dynamics before, during, and after the Inca Empire*

We also conducted an analysis focusing on ancient Inca populations and those incorporated into the Inca Empire over time (Figure 4, panel b). During the Inca expansion (≈512-418 yBP), two haplogroups appear to exhibit pronounced frequency shifts: haplogroup A increased from 16.9% to 26.1%, whereas haplogroup B declined from 20.5% to 6.5%. After the empire’s fall, haplogroup A continued to rise, reaching 31.6% around 300 yBP, while haplogroup C remained the most frequent throughout (45.8-52.5%). However, these patterns cannot be attributed solely to the expansion, as the ancient Inca sample is itself dominated by haplogroups B (55.5%) and D (20%), suggesting that the observed shifts likely reflect differential incorporation of surrounding populations and/or temporal sampling rather than a uniform Inca-specific effect.

*Brazil*

The volume of ancient DNA data from Brazil has expanded substantially in recent years, encompassing samples ranging from the Early Holocene to the colonial period (Moreno-Mayar et al., 2018; Posth et al., 2018; Cruz Dávalos et al., 2022; Ferraz et al., 2023). Because the Brazilian dataset aggregates samples from geographically distant and culturally distinct contexts that may be temporally proximate (e.g., Amazonian and coastal *Sambaqui* populations), temporal patterns shown here may partly reflect underlying spatial structure.

**Figure 5 -:**
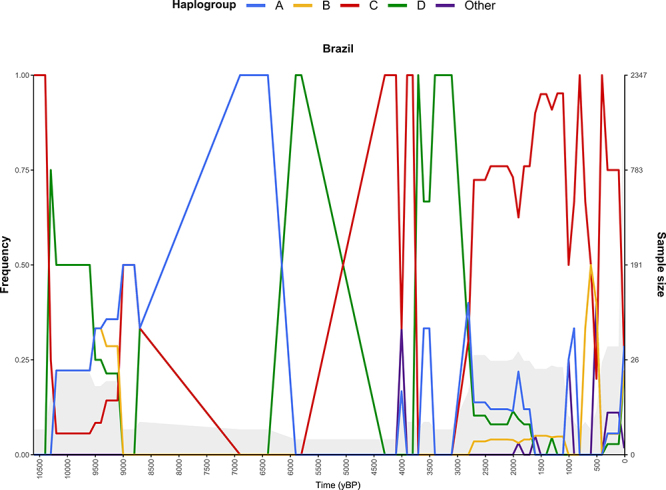
Temporal changes in ancient and modern mtDNA haplogroup frequencies in Brazil. Lines represent the relative frequencies of haplogroups A, B, C, D, and Other, the latter including Native American lineages with unresolved classification or non-Native haplogroups introduced through admixture. The final interval (0 yBP) represents aggregated contemporary populations. Grey shading shows sample size through temporal bins on a compressed power-scaled axis (λ = 0.2) normalized within the region; right-side labels report raw sample sizes (N). Time is in years before present (yBP). Note that the time and sample-size axes are scaled independently.

In Figure 5, we observe a marked predominance of haplogroup D in some of the oldest samples (50-75%; 10,300-9,600 yBP) and between 3,700 and 2,800 yBP (30-100%), followed by the emergence of higher frequencies of haplogroup A between 9,500 and 6,400 yBP (33.3-100%) and 2,800 to 1,900 yBP (12-40%). Haplogroup B appears at notable frequencies primarily in both earlier (10,200-9,100 yBP; 22.2-33.3%) and much later contexts (700-500 yBP; 33.3-50%). Haplogroup C, in contrast, is consistently frequent across multiple temporal intervals, dominating in some of the oldest samples (10,600-10,300 yBP; 9,000-8,700 yBP; 25-100%), re-emerging toward the end of the Middle Holocene (4,300-3,800 yBP; 33.3-100%), and maintaining substantial representation since 2,800 yBP in the Late Holocene (20-100%). In contemporary Brazil (time = 0), all four canonical Native American haplogroups are present at relatively balanced frequencies between themselves, with haplogroup A being the most common (28.7%), followed by C, B, and D (25.6%, 22.2%, and 21.8%, respectively). Accordingly, Figure 5 is interpreted as a heuristic summary of large-scale temporal tendencies in maternal lineage representation, rather than as evidence of continuity or stability within specific Brazilian populations.

We noted unusually high proportions of lineages categorized as “Other” at ≈4,000, 1,000, and 500 yBP. This pattern is primarily attributable to sequences from Santos et al. (1996), in which several individuals could not be confidently assigned to any of the four canonical haplogroups (A-D) due to reliance on short HVR-I marker alone; these lineages were designated “groups V and VI” and most likely represent now-recognized sublineages of A-D that were indistinguishable with the resolution available at the time. A second study contributing to elevated frequencies in the “Other” category between ≈300-100 yBP is Leite et al. (2014). In this case, however, the effect is genuine, reflecting the identification of four individuals bearing African L-lineages in Guajajara village cemeteries, a signal interpreted as the result of admixture with African or Afro-descendant populations during the colonial period, or possibly of enslaved Africans interred within Indigenous burial grounds (Leite *et al*., 2014).

*Sambaqui* populations (ancient shell-mound-building Indigenous societies; Ferraz et al., 2023) are well represented in our ancient dataset, spanning from the oldest sample at Capelinha (10,556-10,290 yBP) to the youngest at Sambaqui Limão (528-479 yBP). Pooling 52 individuals across regions and periods, mean mtDNA haplogroup frequencies were 59.6% C, 13.5% A, 13.5% D, 5.8% B, and 7.6% “Other”. The elevated frequency of haplogroup C is also evident in Figure 5 for the interval 2,800-700 yBP.

### Population structure and isolation-by-distance analyses

After filtering out populations with sample sizes smaller than ten individuals, a total of 256 Native American populations were retained for the analyses. AMOVA showed that most genetic variation resides within populations (73.3%), with significant differentiation among populations within subcontinents (F_SC_ = 0.22; 20.7%, *P* < 0.00001) and a smaller, yet significant, component among subcontinental-scale groupings (F_CT_ = 0.06; 6.0%, *P* < 0.00001). Overall structure was relatively high (F_ST_ = 0.266; *P* < 0.00001), indicating pronounced local differentiation against a backdrop of shared continental ancestry (Table S3).

To further evaluate whether this structure was homogeneous across regions, we conducted subcontinent-specific AMOVAs. In all subcontinents, the majority of genetic variation remained within populations (≈75-80%), indicating a broadly consistent pattern of intrapopulation diversity (Table S3). Levels of among-population differentiation were moderate and comparable across North America (F_ST_ = 0.247), Central America (F_ST_ = 0.241), and South America (F_ST_ = 0.202) (all *P* < 0.00001), with slightly lower differentiation in South America (Table S3). In contrast, the Caribbean showed low and non-significant differentiation (FST = 0.041, *P* = 0.192), likely reflecting limited population sampling. Overall, subcontinental differences in genetic structure are modest and consistent with the continental signal observed in the hierarchical AMOVA.

Mantel tests revealed a significant but weak positive correlation between pairwise F^ST^ and geographic distance at the continental scale (r = 0.189, *P* < 0.0001), indicating a detectable isolation-by-distance signal. Significant correlations were also observed for ecoregional (r = 0.113, *P* = 0.0019) and linguistic (r = 0.055, *P* < 0.0001) dissimilarity when evaluated independently, although effect sizes were substantially smaller than that observed for geographic distance (Table S4).

Multiple regression on distance matrices (MRM) clarified that geographic distance was the only predictor retaining a significant independent association with genetic differentiation when all variables were considered jointly (β_GEO ≈ 1.37 × 10⁻⁵, *P* < 0.0001). In contrast, ecoregional and linguistic effects lost significance after controlling for geography (Table S5). Importantly, all continental models explained only a small proportion of the total variance (R² ≈ 0.03-0.04, corresponding to ≈3-4% of the observed genetic differentiation), indicating that these predictors account for only a limited fraction of the observed genetic differentiation.

Subcontinental analyses revealed marked heterogeneity. In North America, both geographic distance and linguistic affiliation retained significant independent effects in multiple MRM models, resulting in higher overall explanatory power for the combined model (R² ≈ 0.12, ≈12% of the variance), which nonetheless remains modest in absolute terms. In South America, associations were weak, with geographic distance showing only a marginal independent effect in the full model (R² ≈ 0.009, <1% of the variance), and no significant contributions from linguistic or ecoregional predictors. No robust associations were detected in Central America or the Caribbean, likely reflecting limited statistical power and reduced variability in linguistic and ecoregional composition within these macro-regions.

## Discussion

### Marked expansion of aDNA recovery from warm-humid environments

The results reveal a marked increase in ancient DNA samples originating from tropical and subtropical regions (Figure 1, Table S2). Recent methodological breakthroughs have revolutionized ancient DNA recovery in hot and humid settings, driving a surge of successful studies across the American continent (Roca-Rada et al., 2020; Nieves-Colón, 2022; Llamas et al., 2017). Critically, short-read high-throughput sequencing (next-generation sequencing) has been the most transformative enabler of aDNA, allowing the parallel sequencing of millions of ultrashort molecules and superseding PCR-based approaches (Orlando et al., 2021; Llamas *et al*., 2017). Equally critical, rigorous contamination-control standards and authenticity criteria established in the high-throughput sequencing era (Llamas *et al*., 2017) have ensured the reliability of these new datasets, integrating field archaeology and molecular protocols to maximize endogenous DNA recovery. Optimized silica-based extraction protocols using chaotropic buffers now efficiently retain ultrashort molecules (≥25-35 bp) while reducing co-extracted inhibitors (Rohland et al., 2018), and dense substrates, particularly the otic capsule of the petrous bone, yield markedly higher endogenous DNA fractions, reaching up to ≈65-177× higher recovery rates compared with other petrous regions, even under warm or arid conditions (Pinhasi et al., 2015; Llamas *et al*., 2017). Minimally destructive methods targeting the dental cementum further preserve irreplaceable specimens while maintaining high endogenous yields (Harney et al., 2021).

In parallel, single-stranded DNA library preparation (ssDNA 2.0) and its robust T4-ligase implementation have enhanced the recovery of highly fragmented molecules and enabled automation compatible with high-throughput sequencing (Gansauge et al., 2017, 2020). Moreover, cost-effective in-solution hybridization capture panels covering hundreds of thousands to over one million SNPs have made genome-scale analyses feasible even for samples with minimal endogenous content (Rohland et al., 2022; Llamas et al., 2017). Collectively, these innovations underpin the recent expansion of tropical aDNA datasets in the Americas, exemplified by genome-wide studies from the Caribbean (Nägele et al., 2020; Fernandes et al., 2021) and multiple regions of Brazil (Ferraz et al., 2023).

### Continuity and discontinuity

*Homo sapiens*, when observed through time and space since the initial arrival on the American continent, exhibit patterns of genetic and cultural continuity and discontinuity. Some events are so consequential that they can cause severe disruptions, both genetic and cultural, across the entire continent, such as the arrival of Europeans in the fifteenth century. Within this framework, post-contact genetic discontinuities are captured as shifts in maternal lineage composition, including the emergence or increase of non-Native mtDNA haplogroups represented in the “Other” category, rather than as direct evidence of complete population replacement. Other discontinuity-inducing events are more specific and can cause both genetic and cultural discontinuities simultaneously, or just one or the other. The interpretation of continuities will also depend on the type of data that is being analyzed. In genetics, different markers can lead to different interpretations, like 10,000 years of regional genetic continuity that Lindo et al. (2017) observed in the North American Northwest Coast using genome-wide sequences, despite regional shifts in mtDNA haplogroups. In other situations, the same event can be marked by a cultural discontinuity with a genetic continuity, as occurred with the Wari when their empire collapsed (Kemp et al., 2009).

Beyond intergroup domination, conflict, and colonialism, environmental variability may also have precipitated demographic reconfigurations that could register as genetic discontinuities. In the Eastern Arctic, population turnover from Dorset to Thule plausibly intersected with paleoclimatic fluctuations associated with the Medieval Climate Anomaly (MCA) around 1,000 to 700 yBP and subsequent Little Ice Age (LIA) cooling after about 650 yBP, well-established North Atlantic climatic phases that may have favored the rapid expansion of Thule ancestors and the decline of Dorset populations (Friesen et al., 2020). In Mesoamerica, high-resolution speleothem records from the northern Yucatán document multi-year droughts between 1,079 and 929 yBP, which may have stressed Classic Maya societies and contributed to regional demographic change (James et al., 2025). Along the Peruvian coast, the El Niño-Southern Oscillation (ENSO), a coupled ocean-atmosphere pattern in the tropical Pacific, appears to have intensified, with a shift to stronger and more frequent events after about 5,800 yBP and especially after about 3,000 yBP, plausibly amplifying flood and drought risks that could disrupt settlement and subsistence systems (Sandweiss, 2003). In Amazonia, multiple proxies indicate drier conditions during the Early to Mid-Holocene, roughly 8,000 to 4,000 yBP, with ecotonal forest-savannah shifts and elevated fire activity in several regions, processes that could fragment habitats and reshape local population dynamics (Mayle and Power, 2008). Farther south, archaeological sequences in southern Mendoza register an occupational hiatus between about 6,000 and 4,000 yBP that the authors attribute to increased aridity, consistent with environmentally driven demographic contractions (Gil et al., 2005). At a continental scale, summed radiocarbon probability curves reveal downturns centered on approximately 8,600 to 6,600 yBP and 2,500 to 2,000 yBP, which may reflect climate-linked reductions in population densities across parts of South America (Riris and Arroyo-Kalin, 2019). These examples are included to provide readers with broader environmental context in this continent-wide synthesis; quantitatively testing climate-demography-genetics links would require dedicated spatiotemporal modeling and is beyond the scope of this review.

The spatial and temporal patterns presented in Figures 2 and 3 illustrate how demographic processes have shaped Native American mitochondrial diversity. The interpolated maps of present-day haplogroup frequencies (Figure 2) reflect the cumulative outcome of long-term population structure, migration, and genetic drift across the continent. However, the temporal reconstructions based on ancient, historical, and present-day datasets (Figure 3a-d ) show that haplogroup frequencies seem to have fluctuated through time within different subcontinental regions. Taken together, these results indicate that the contemporary geographic distribution of Native American mitochondrial lineages should be interpreted as the product of a dynamic demographic history rather than as a static reflection of the earliest population structure established during the initial peopling of the American continent.

### Peopling of the Caribbean

In the previous study (Bisso-Machado and Fagundes, 2021), Central America and the Caribbean were analyzed jointly. In the present study, we examine them separately, given the recent 269.8% increase (Table S2) in aDNA studies in the region (Schroeder et al., 2018; Nägele et al., 2020; Nieves-Colón et al., 2020; Fernandes et al., 2021; Forbes-Pateman et al., 2022). Current aDNA evidence indicates that the Caribbean was first settled during the Archaic (Lithic) Age by groups related to Central and northern South America beginning ≈6,000 years ago, with securely dated sites by ≈5,000 cal BP and “leapfrog” island dispersals (Nägele *et al*., 2020; Forbes-Pateman *et al*., 2022). A second, transformative migration unfolded in the Ceramic Age: genome-wide data show that a largely homogeneous population related to Arawak speakers of northeast South America spread through the Lesser Antilles into the Greater Antilles ≥1,700 years ago, replacing most Archaic ancestry and forming small, highly connected island populations (Fernandes *et al*., 2021; Forbes-Pateman *et al*., 2022).

Individual genomes refine this picture, the Lucayan Taíno from the Bahamas clusters with Arawakan speakers from the Amazon (Orinoco region), supporting a northern South American source for Greater Antillean lineages (Schroeder et al., 2018). Mitochondrial data from precontact Puerto Rico, dominated by A2 and C1 (notably C1b2), likewise indicate primarily Amazonian/South American origins and document continuity of specific haplotypes into present-day islanders (Nieves-Colón et al., 2020). Syntheses further note no genetic evidence for substantial inputs from Florida/southern North America and clarify that earlier proposals of two distinct migrations into Cuba are not supported by broader datasets (Fernandes et al., 2021; Forbes-Pateman et al., 2022). The results of the present study (Figure 3, panel c) suggest a shift in haplogroup composition coinciding with the estimated timing of Archaic ancestry replacement, characterized by an increase in haplogroup A and a decrease in haplogroup D.

### The cultural succession in the Arctic and mtDNA

The pattern observed in Figure 4 (panel a), showing the alternation and eventual dominance of haplogroups A and D from the earliest Paleo-Eskimo to the present Inuit populations, mirrors the demographic succession documented for the Arctic cultures. Early prevalence of haplogroup D, especially sublineages D2a and D2a1, corresponds to the genetic profile of the Saqqaq and Dorset Paleo-Eskimos, who descended from a single Siberian migration around 6,000 BP (≈ 4,000 BCE) and maintained genetic continuity for more than four millennia (Raghavan et al., 2014). Archaeological evidence from the early Denbigh Flint Complex in Alaska, dated to 5,500-4,000 cal BP (≈ 3,550-2,050 BCE), represents the earliest phase of the Arctic Small Tool tradition (Harritt, 1998), which subsequently expanded eastward, giving rise to the Saqqaq and Pre-Dorset cultures between ≈4,800-2,800 BP (≈ 2,800-800 BCE) (Friesen, 2004).

The sharp increase and sustained dominance of haplogroup A from ≈1,700 BP (≈ 250 CE) onward coincides with the initial demographic shifts that preceded the emergence of Neo-Inuit (formerly termed Neo-Eskimo) groups in the Bering Strait region. These populations, associated with the Birnirk and Punuk cultures (6th-7th centuries CE; ≈1,450-1,300 BP), interacted and ultimately gave rise to the Thule culture, the direct ancestors of modern Inuit (Raghavan et al., 2014; Sørensen and Diklev, 2022). The subsequent demographic replacement, dated to ≈1,150-700 BP (≈ 800-1,250 CE), is further supported by the absence of matrilineal gene flow between Dorset and Thule populations (Raghavan *et al*., 2014). The predominance of haplogroup A in present-day Inuit (≈ 71.6%), as shown in Figure 4 (panel a), thus reflects the demographic success of Thule descendants following their rapid eastward expansion after ≈700 BP (≈ 1,250 CE), whereas residual D frequencies likely represent Neo-Inuit D3a2a variants rather than surviving Paleo-Inuit ancestry. Overall, these results demonstrate that the mitochondrial lineage turnover inferred from Figure 4 (panel a) parallels the well-documented cultural succession from Dorset to Thule, marking a profound reconfiguration of the maternal genetic landscape of the circumpolar Arctic.

### Genetic continuity and demographic transitions through Andean cultural periods

Our quantitative synthesis of haplogroup frequencies across 4,300-300 BP (Figure 4, panel b) reveals diachronic trends that closely parallel both archaeological periodization and paleoclimatic reconstructions for the Central Andes. The Early Horizon and Early Intermediate Period (*ca*. 2,850-1,350 BP) are dominated by haplogroup D in Paracas (77%) but show its relative decline in Nasca (53%) alongside a pronounced rise of C (32%) and emerging A lineages, consistent with the demographic reorganization and increased inter-valley interaction that accompanied progressive aridification and the eventual collapse of the Nasca cultural system (Fehren-Schmitz et al., 2010). During the Middle Horizon (ca. 1,350-950 BP), Wari and Tiwanaku populations display a distinct shift toward haplogroups B (43-50%) and C (≈30%) with minimal D, matching the genetic homogenization produced by intensified mobility and state integration under Wari-Tiwanaku hegemony (Fehren-Schmitz *et al*., 2011, 2014).

Fehren-Schmitz et al. (2014) explicitly linked these demographic adjustments to El Niño-Southern Oscillation-driven climatic oscillations, droughts in the highlands and episodic floods on the coast, that triggered sequential migrations upslope around 640 CE (≈1,350 yBP) and subsequent back-migrations toward the coast near 1200 CE (≈750 yBP). The Late Intermediate Period (950-500 BP) maintains this homogenized pattern, as observed in Ychsma and Lambayeque, where B and C dominate and D remains residual (Russo et al., 2018). In the Late Horizon and early Colonial phase (≤ 500 BP), the gradual rise of A and decline of B within Inca and Chachapoya-Inca samples (A ≈ 7-18%; B ≈ 55%) coincide with large-scale state relocations and subsequent population collapse after European contact (Fehren-Schmitz *et al*., 2014; Lindo et al., 2018 b ). Collectively, these spatiotemporal fluctuations confirm that Andean mitochondrial variation reflects a pattern of long-term genetic continuity punctuated by episodes of environmentally and politically driven mobility, rather than abrupt exogenous population replacements (Llamas et al., 2016).

### Temporal dynamics of mitochondrial diversity in Holocene Brazil

Due to the limited number of available sequences, broad inferences about ancient mtDNA distributions in Brazil remain uncertain, as sharp frequency shifts over time likely reflect sampling noise rather than true demographic events. More robust patterns emerge only at two temporal scales: the Early Holocene and the Late Holocene (especially from ≈2,700 yBP to the present). The earliest individuals, attributed to Paleoamerican contexts, exhibit a predominance of haplogroup D, followed by the appearance of A and B, consistent with the ancestral composition of the first South American settlers (Posth et al., 2018; Moreno-Mayar et al., 2018). A clearer demographic signal reappears after ≈2,700 yBP, when haplogroup C becomes dominant, particularly among *Sambaqui* populations such as *Jabuticabeira* II, and remains frequent throughout later periods (Ferraz et al., 2023). This persistence of C, together with the gradual rise of A and the decline of D, coincides with major environmental and cultural transformations in coastal Brazil, including sea-level regression, regional cooling, and the inland expansions of ceramic-producing and agricultural groups (Ferraz *et al*., 2023; Iriarte et al., 2017; Castro e Silva et al., 2020, 2022). While most *Sambaqui* sites disappeared after ≈2,200 yBP, genetic continuity and interaction between coastal and inland groups suggest population reorganization rather than abrupt replacement (Ferraz *et al*., 2023), shaping the mitochondrial landscape of Late Holocene Brazil.

### Continental structure

The AMOVA results are consistent with previous findings (Bisso-Machado and Fagundes, 2021). Most mitochondrial variation is retained within populations (73.3% here vs 72.29-74.43%), with substantial differentiation among populations within subcontinents (F_SC_ = 0.22; 20.7% here vs 15.37-18.88%) and a smaller, yet significant, component among subcontinents (F_CT_ = 0.06; 6.0% vs 7.71-10.44%). The overall level of population structure (F_ST_ = 0.266) closely matches the mtDNA range reported by Bisso-Machado and Fagundes (2021; F_ST_ = 0.256-0.277) across geographic, linguistic, and ecoregional partitions. Subcontinent-specific AMOVAs further show that, although the magnitude of among-population differentiation varies across regions, the predominance of within-population variance is consistently maintained. Together, these parallels indicate that the expanded dataset recapitulates the same hierarchical structure, while allowing for quantitative regional heterogeneity. 

At the same time, the relatively high levels of local differentiation typical of Native American populations warrant caution when interpreting genetic continuity and discontinuity. Strong population structure and genetic drift can generate substantial temporal fluctuations in haplogroup frequencies, potentially obscuring signals of lineage persistence or loss in regional time-series analyses. Such patterns may arise because genetic drift can alter lineage frequencies through time, producing differentiation even among temporally separated samples from the same broader population. In addition, historical admixture events, particularly when the contributing source population is unsampled, may generate genetic patterns that complicate the interpretation of temporal differences among samples (McKenna et al., 2024).

Compared with the earlier dataset, which showed a weaker correlation between mtDNA differentiation and geographic distance (r = 0.049, *P* = 0.007), the expanded dataset reveals a more readily detectable geographic signal (Mantel r = 0.189, *P* < 0.0001; MRM R² ≈ 0.03-0.04). Importantly however, the proportion of variance explained remains low, reinforcing that geographic distance alone is a limited predictor of mtDNA structure when summarized at broad continental scales. This increase in statistical detectability likely reflects broader population coverage and the exclusion of under-sampled groups, which reduce noise and yield a clearer population-level signal of isolation by distance. Consistent with this interpretation, subcontinental analyses indicate that the strength of geographic effects varies across regions.

Taken together, these results suggest that although spatial and cultural variables can exhibit statistically detectable associations with mtDNA differentiation, their explanatory power is limited. This pattern is consistent with a mitochondrial landscape shaped by complex demographic histories, including drift, founder effects, and population turnover, rather than by stable spatial or cultural boundaries alone.

## Conclusion

This review compiles all available data on ancient and contemporary mtDNA in Native American populations, providing a continent-wide synthesis on approximately 16,000 years of maternal genetic history in America. By integrating population structure analyses with time-ordered haplogroup frequency trajectories, this study moves beyond regional case studies and offers a unified framework for evaluating patterns of genetic continuity and discontinuity across the continent. 

Our results show that Native American mitochondrial variation is largely local in scale: most diversity resides within populations, subcontinental structure is modest, and geographic distance explains only a small fraction of differentiation (3-4%), indicating a consistent yet weak isolation-by-distance signal. Against this backdrop, temporal haplogroup frequency series reveal recurrent and replicable regional dynamics, including the rise of haplogroup A in Neo-Inuit contexts, the Archaic-to-Ceramic transition in the Caribbean, and the long-term prominence of haplogroup C among eastern South American coastal groups. Together, these patterns highlight a mitochondrial landscape shaped by both persistence of maternal lineages and repeated episodes of regional reorganization.

Because haplogroup frequencies alone cannot distinguish between population continuity and replacement, we outline three practical directions to turn descriptive patterns into tested demographic scenarios: (i) sub-haplogroup analyses with regionally time-sliced networks to resolve lineage-level dynamics; (ii) formal temporal modeling to detect and date inflection points in haplogroup trajectories; and (iii) targeted sampling of admixed populations to trace the persistence of Native maternal lineages beyond post-contact bottlenecks.

Finally, it is important to emphasize that the modern populations represented here reflect groups that survived European contact and therefore capture only a subset of Native mitochondrial diversity. Extending analysis to admixed populations, and anchoring mitochondrial trajectories to well-dated archaeological, cultural and climatic frameworks, will be essential to clarify when continuity prevailed and when demographic reconfiguration reshaped the maternal genetic landscape of America. In this sense, the dataset assembled here and the general analyses conducted provide not only a synthesis of existing knowledge, but an important reference point for future demographic modeling of the initial peopling and subsequent re-shaping of the American continent. 

## Supplementary Material

The following online material is available for this article:

Table S1 - Information for mtDNA in modern samples, including sample size, haplogroup frequency, linguistic and geographical information.

Table S2 - Information for mtDNA in ancient samples, including sample size, haplogroup frequency, geographical and archaeological information.

Table S3 - Results of hierarchical (continental) and subcontinent-specific AMOVA based on haplogroup frequencies in American populations.

Table S4 - Results of Mantel tests assessing the correlation between pairwise mitochondrial genetic differentiation (F_ST_) and geographic distance, linguistic, and ecoregional dissimilarity at continental and subcontinental scales.

Table S5 - Multiple regression on distance matrices (MRM) evaluating geographic, linguistic, and ecoregional effects on mitochondrial genetic differentiation.

## Data Availability

All original data sources are documented in Bisso-Machado and Fagundes (2021) and in the Supplementary Material of this article.
