# Supplementary material for: Continuity and discontinuity in Native American populations: Insights from ancient and modern mitochondrial DNA
Source: Genet Mol Biol. 2026 Jul 20;49(Suppl 1):e20250248. doi: 10.1590/1678-4685-GMB-2025-0248 (PMC13403483; doi:10.1590/1678-4685-GMB-2025-0248)
Supplement: Table S2 - [file 1415-4757-GMB-49-s1-e20250248-s2.pdf]

Genetics and Molecular Biology
https://doi.org/ 10.1590/1678-4685-GMB-2025-0248

Supplementary Material to "Continuity and discontinuity in Native American populations: insights from ancient and modern mitochondrial DNA"

Table S2 - Information for mtDNA in ancient samples, including sample size, haplogroup frequency, geographical and archaeological information.

The table is available at the following DOI link:

https://doi.org/10.6084/m9.figshare.30520985
