# Supplementary material for: Continuity and discontinuity in Native American populations: Insights from ancient and modern mitochondrial DNA
Source: Genet Mol Biol. 2026 Jul 20;49(Suppl 1):e20250248. doi: 10.1590/1678-4685-GMB-2025-0248 (PMC13403483; doi:10.1590/1678-4685-GMB-2025-0248)
Supplement: Table S3 - [file 1415-4757-GMB-49-s1-e20250248-s3.pdf]

Genetics and Molecular Biology

https://doi.org/ 10.1590/1678-4685-GMB-2025-0248

Supplementary Material to "Continuity and discontinuity in the American continent: insights from ancient and modern mitochondrial DNA"

Table S3 – Results of hierarchical (continental) and subcontinent-specific AMOVA based on haplogroup frequencies in American populations.

AMOVA level	# pop	# ind	Source of variation	d.f.	Sum of squares	Variance components	% variation	F-statistics	F-statistics *P-value*
**Continental**	256	23,067	Among subcontinents	3	314.001	0.02265	6	FCT = 0.0599	< 0.00001
			Among populations within subcontinents	252	1,808.80	0.07803	20.65	FSC = 0.2197	< 0.00001
			Within populations	22,811	6,321.90	0.27714	73.35	FST = 0.2665	< 0.00001
**Subcontinental**									
North America	67	9,058	Among populations	66	729.409	0.08212	24.67	FST = 0.2467	< 0.00001
			Within populations	8,991	2,254.77	0.25078	75.33	–	–
Central America	13	932	Among populations	12	61.467	0.0734	24.14	FST = 0.2414	< 0.00001
			Within populations	919	211.447	0.2307	75.86	–	–
Caribbean	2	55	Among populations	1	0.52	0.01238	4.12	FST = 0.0412	0.19218
			Within populations	53	15.262	0.28796	95.88	–	–
South America	174	13,022	Among populations	173	1,017.41	0.0755	20.17	FST = 0.2017	< 0.00001
			Within populations	12,848	3,839.89	0.2988	79.83	–	–

The continental AMOVA was hierarchical, with populations nested within subcontinents (North America, Central America, Caribbean, and South America). In this model, FCT quantifies genetic differentiation among subcontinents, FSC represents the average differentiation among populations within subcontinents, and FST measures overall population differentiation across the continent. Subcontinent-specific AMOVAs were conducted separately for each region and estimate genetic differentiation among populations within each subcontinent using FST. Significance of variance components and fixation indices was assessed by permutation tests. Note: The total number of populations and individuals included in each analysis may differ from the full dataset, as only populations with sample sizes ≥10 individuals were retained to minimize stochastic sampling effects associated with small sample sizes. # pop indicates the number of populations, # ind indicates the number of individuals, and d.f. denotes degrees of freedom.
