# Supplementary material for: Continuity and discontinuity in Native American populations: Insights from ancient and modern mitochondrial DNA
Source: Genet Mol Biol. 2026 Jul 20;49(Suppl 1):e20250248. doi: 10.1590/1678-4685-GMB-2025-0248 (PMC13403483; doi:10.1590/1678-4685-GMB-2025-0248)
Supplement: Table S4 - [file 1415-4757-GMB-49-s1-e20250248-s4.pdf]

Genetics and Molecular Biology
https://doi.org/ 10.1590/1678-4685-GMB-2025-0248

Supplementary Material to "Continuity and discontinuity in Native American populations: insights from ancient and modern mitochondrial DNA"

Table S4 – Results of Mantel tests assessing the correlation between pairwise mitochondrial genetic differentiation (F_{ST}) and geographic distance, linguistic, and ecoregional dissimilarity at continental and subcontinental scales.

Scale	Model	Number of populations	Mantel *r*	*P-value*
Continental	F_{ST} ~ Geographic distance	256	0.1890	0.0001
	F_{ST} ~ Ecoregion	256	0.1126	0.0019
	F_{ST} ~ Language	238	0.0549	0.0001
Subcontinental				
North	F_{ST} ~ Geographic distance	67	0.3253	0.0001
	F_{ST} ~ Ecoregion	67	0.1563	0.0001
	F_{ST} ~ Language	67	0.1768	0.0001
Central	F_{ST} ~ Geographic distance	13	0.1100	0.2656
	F_{ST} ~ Language	13	0.1705	0.0898
South	F_{ST} ~ Geographic distance	174	0.0605	0.0823
	F_{ST} ~ Ecoregion	174	0.0103	0.4122
	F_{ST} ~ Language	157	0.0287	0.0574

P-values were obtained by permutation tests (10,000 permutations). Only populations with sample sizes ≥10 individuals were included. Caribbean populations were not tested due to insufficient sample size (N = 2). Ecoregional dissimilarity was not evaluated in Central America due to limited variation in ecoregional categories within this region. Linguistic analyses include only populations with assigned linguistic affiliation; therefore, N may vary across models.
