# Supplementary material for: Continuity and discontinuity in Native American populations: Insights from ancient and modern mitochondrial DNA
Source: Genet Mol Biol. 2026 Jul 20;49(Suppl 1):e20250248. doi: 10.1590/1678-4685-GMB-2025-0248 (PMC13403483; doi:10.1590/1678-4685-GMB-2025-0248)
Supplement: Table S5 - [file 1415-4757-GMB-49-s1-e20250248-s5.pdf]

Genetics and Molecular Biology
https://doi.org/ 10.1590/1678-4685-GMB-2025-0248

Supplementary Material to "Continuity and discontinuity in Native American populations: insights from ancient and modern mitochondrial DNA"

Table S5 – Multiple regression on distance matrices (MRM) evaluating geographic, linguistic, and ecoregional effects on mitochondrial genetic differentiation.

Scale	Model	Predictor	N populations	β Coefficient	*P-value*	R^2
Continental	FST ~ Geographic distance	GEO	256	0.00001	0.0001	0.03573
	FST ~ Ecoregion	ECO	256	0.04260	0.0023	0.01268
	FST ~ Language	LANG	238	0.05671	0.0001	0.00301
	FST ~ Geographic distance + Ecoregion + Language	GEO	238	0.00001	0.0001	
	FST ~ Geographic distance + Ecoregion + Language	ECO	238	0.00297	0.8442	0.04457
	FST ~ Geographic distance + Ecoregion + Language	LANG	238	0.01690	0.1607	
Subcontinental						
North America	FST ~ Geographic distance	GEO	67	0.00005	0.0001	0.10580
	FST ~ Ecoregion	ECO	67	0.07132	0.0001	0.02443
	FST ~ Language	LANG	67	0.14129	0.0001	0.03125
	FST ~ Geographic distance + Ecoregion + Language	GEO	67	0.00004	0.0002	
	FST ~ Geographic distance + Ecoregion + Language	ECO	67	0.01197	0.3376	0.11607
	FST ~ Geographic distance + Ecoregion + Language	LANG	67	0.07914	0.0020	
Central America	FST ~ Geographic distance	GEO	13	0.00004	0.5503	0.01211
	FST ~ Language	LANG	13	0.05313	0.1700	0.02906
South America	FST ~ Geographic distance	GEO	174	0.00001	0.1397	0.00366
	FST ~ Ecoregion	ECO	174	0.00436	0.8553	0.00011
	FST ~ Language	LANG	157	0.02129	0.1131	0.00082
	FST ~ Geographic distance + Ecoregion + Language	GEO	157	0.00001	0.0401	
	FST ~ Geographic distance + Ecoregion + Language	ECO	157	-0.00337	0.9048	0.00877
	FST ~ Geographic distance + Ecoregion + Language	LANG	157	0.00948	0.5174	

Results of multiple regression on distance matrices (MRM) assessing the independent and joint effects of geographic distance (GEO), linguistic affiliation (LANG), and ecoregional dissimilarity (ECO) on pairwise mitochondrial genetic differentiation (FST) at continental and subcontinental scales. Regression coefficients (β) and P-values are reported for each predictor, while R^2 corresponds to the proportion of variance explained by the overall model and is therefore identical across predictors within the same model. Statistical significance was evaluated using permutation tests (10,000 permutations). Only populations with sample sizes ≥10 individuals were included. Ecoregional dissimilarity was not evaluated in Central America due to limited variation in ecoregional categories, and Caribbean populations were not analyzed due to insufficient sample size (N = 2). Linguistic analyses include only populations with assigned linguistic affiliation; therefore, N may vary across models.
